# Effect of the COVID-19 Pandemic on Resting-State Brain Activity in Individuals with Tinnitus

**DOI:** 10.3390/brainsci14020174

**Published:** 2024-02-12

**Authors:** W. Wiktor Jedrzejczak, Elżbieta Gos, Malgorzata Ganc, Danuta Raj-Koziak, Piotr H. Skarzynski, Henryk Skarzynski

**Affiliations:** 1 Institute of Physiology and Pathology of Hearing, Mochnackiego 10, 02-042 Warsaw, Poland; e.gos@ifps.org.pl (E.G.); m.ganc@ifps.org.pl (M.G.); d.koziak@ifps.org.pl (D.R.-K.); p.skarzynski@csim.pl (P.H.S.); skarzynski.henryk@ifps.org.pl (H.S.); 2 World Hearing Center, Mokra 17, Kajetany, 05-830 Nadarzyn, Poland; 3 Institute of Sensory Organs, Mokra 1, Kajetany, 05-830 Nadarzyn, Poland; 4 Heart Failure and Cardiac Rehabilitation Department, Faculty of Medicine, Medical University of Warsaw, Kondratowicza 8, 03-242 Warsaw, Poland

**Keywords:** COVID-19, tinnitus, THI, EEG

## Abstract

This study looked at the possible effect of the COVID-19 pandemic on individuals who came to our clinic seeking relief from tinnitus. The performance of the subjects during the COVID-19 pandemic was compared with similar individuals who came to our clinic before the pandemic began. The study involved 50 adults with chronic tinnitus, made up of a study group (24 subjects tested during the COVID-19 pandemic of 2020–2021) and a control group before the pandemic began (26 subjects tested from 2013 to 2017). None of the 24 reported having contracted COVID-19. Data collection involved the Tinnitus Handicap Inventory (THI) questionnaire, audiological tests, and quantitative electroencephalography (qEEG). In terms of THI scores, there were no statistically significant differences between the two groups. However, with regard to qEEG, some changes were observed, with significant decreases in alpha and beta band activity in the study group compared to the control group, particularly over the auditory cortex. We conclude that COVID-19 did not have a discernible impact on the general well-being of individuals with tinnitus. However, it did appear to alter brain activity, specifically in the alpha and beta bands over the auditory cortex, and these reults warrant further investigation.

## 1. Introduction

The subjective experience of tinnitus, the perception of a sound that is not externally present, remains complex and is not fully understood [1]. Recent studies lean toward the idea that there is some deficiency in central neural processing rather than in peripheral hearing decline [2]. There is some compelling evidence suggesting a link between tinnitus and the psychological state of the individual [3]. Stress and anxiety have been identified as factors exacerbating tinnitus, so interventions aimed at reducing stress levels show some effectiveness in managing symptoms [4].

Recently, we have seen the pervasive impact of the COVID-19 pandemic, which seems to extend beyond the realm of physical health to include heightened uncertainty about the future, even among those fortunate enough not to experience severe symptoms [5]. This uncertainty has probably led to increased stress and anxiety across the population in general.

In the present study, our focus is not on the direct impact of COVID-19 infection on individuals with tinnitus but rather on broader repercussions during the pandemic period. Possible factors include psychological stress induced by issues like isolation, fear of infection, and long-term consequences. Individuals with tinnitus, who are often already grappling with psychological and psychiatric challenges, are a group particularly vulnerable to the compounding effects of the pandemic [6,7]. Studies have indicated a surge in tinnitus symptoms and a higher prevalence of tinnitus among those infected with the virus [6,7]. The pandemic no doubt exacerbated the challenges faced by tinnitus sufferers, disrupting their routines and limiting their access to healthcare [5,8,9].

While there is no objective diagnostic method for subjective tinnitus [10], resting-state electroencephalography (EEG), particularly when analyzed at the group level, has emerged as a promising measure for people with tinnitus [11,12]. EEG studies, together with magnetoencephalography (MEG), have consistently found associations between tinnitus and alterations in neural activity—reflected in decreased alpha and increased delta power in the temporal regions, as well as heightened gamma power in the auditory cortex [13,14].

The COVID-19 pandemic has significantly complicated the process of recording electrical brain activity, particularly the isolation measures required [15]. Other repercussions of the pandemic include less access to participants, difficulty in collecting data, and limited recruitment [16]. Be that as it may, some EEG work performed during prolonged isolation periods has reported that there seems to be decreased alpha power in the resting-state EEG [17]. Other studies have identified an association between isolation during the pandemic and increased depressive symptoms [18].

The rationale for this study builds on the limited research base of the effect of COVID-19 on tinnitus, the paucity of EEG studies during the pandemic, and the absence during the pandemic of studies combining EEG measures with tinnitus. We sought to address this gap by looking at whether COVID-19 had an impact on the daily lives of individuals with tinnitus and, more particularly, whether we could see any measurable changes in resting EEG patterns utilizing quantitative EEG (qEEG). Our hypothesis was that the tinnitus sufferers would report some decline in their mood during the pandemic period and that it would be paralleled with some changes in the qEEG, most likely as a decrease in alpha band power, as has been seen in some studies investigating prolonged isolation [17].

## 2. Materials and Methods

### 2.1. Participants

This retrospective investigation is based on data from patients referred to our tertiary Ear, Nose, and Throat (ENT) center in Poland for troublesome tinnitus. These patients took part in some of our previous studies involving qEEG [12,19] (although a different subset of subjects was selected for the present study). Their tinnitus was either the primary complaint or secondary to hearing loss. The study created two groups of subjects with tinnitus: 24 individuals admitted to our center during the COVID-19 pandemic of 2020–2021 (the study group), and 26 admitted prior to the onset of the pandemic, in the years 2013–2017 (the control group). The first group was made up of 8 women and 16 men ranging in age from 27 to 73 years (M = 49.7; SD = 13.5); the second comprised 9 women and 17 men aged from 30 to 70 years (M = 49.5; SD = 12.0). None of the participants reported having had COVID-19.

### 2.2. Design

Retrospective screening of patient records was conducted to check for eligibility: age above 18 years, tinnitus duration of at least 6 months, documented hearing thresholds determined through clinical pure tone audiometry, performance of a qEEG, and completion of the Tinnitus Handicap Inventory (THI). The assessment of subjects and recording procedures followed those outlined by Raj-Koziak et al. [19]. 

Based on a previous study that reported changes in qEEG alpha power related to the COVID-19 pandemic [20], we estimated that groups of similar size (i.e., at least 20 subjects) should be sufficient to detect changes in tinnitus sufferers. 

The study protocol received approval from the local ethics committee (approval KB/Oświadczenie nr 8/2022), and informed consent was obtained from all subjects.

### 2.3. Audiological Evaluation

Audiological examination comprised videotoscopy, pure-tone audiometry, impedance audiometry, and matching of tinnitus loudness and pitch. Hearing thresholds were determined for both the right and left ears across 0.125, 0.25, 0.5, 1, 2, 4, and 8 kHz (air conduction) and 0.25, 0.5, 1, 2, and 4 kHz (bone conduction). Assessments were conducted using a Madsen Itera II audiometer (GN Otometrics, Taastrup, Denmar). 

Impedance audiometry utilized a Clarinet middle ear analyzer (Inventis, Padova, Italy), and results were deemed abnormal if the middle ear pressure was lower than −150 mm of water and compliance was less than 0.3 cc.

The loudness and frequency of each patient’s tinnitus were determined by presenting sounds matching those described by the patient. Using an audiometer as the source, pure-tone or narrowband noise was presented over headphones at frequencies ranging from 0.125 to 12.5 kHz at a level 10 dB above the participant’s hearing threshold.

### 2.4. Quantitative EEG Measurements

Quantitative electroencephalography (qEEG) was conducted in a quiet room using a Mitsar 21-channel device (Mitsar Co., Ltd., St. Petersburg, Russia). Patients were seated in comfortable chairs and instructed to remain relaxed and still throughout the recording. Signal acquisition involved an ElectroCap with electrodes arranged according to the international 10–20 system and with electrode impedance maintained below 5 kΩ. Signals were filtered between 0.5 and 50 Hz and digitized at 250 Hz. Win EEG 2.84 (Mitsar Co., Ltd.) software facilitated the analysis.

The EEG signals were obtained during resting-state recordings with eyes open and closed (in random order, 2–3 min each) and were analyzed in terms of weighted averages. To minimize artifacts from muscle and eye movements, the signals underwent independent component analysis and visual assessment. Artifact-free, continuous EEG signals were segmented into 4.096 s epochs using a Hanning time window (with 50% overlaps), and Fast Fourier Transforms (FFTs) were performed. The absolute power of the FFT was analyzed in the frequency ranges of delta (1.5–4 Hz), theta (4–8 Hz), alpha (8–10 Hz), alpha2 (10–12 Hz), low beta (12–15 Hz), mid beta (15–18 Hz), and high beta (18–25 Hz).

### 2.5. Tinnitus Handicap Inventory

The Tinnitus Handicap Inventory (THI), developed by Newman et al. [21] and adapted into Polish by Skarzynski et al. [22], was employed to assess the impact of tinnitus on daily living. Consisting of 25 items, it encompasses aspects related to limitations induced by tinnitus, affective responses, and reactions to tinnitus. Patients respond to each item with a “yes” (scored 4 points), “sometimes” (2 points), or “no” (0 points). The individual responses are summed, with higher scores indicating a greater perceived severity of tinnitus.

### 2.6. Statistical Analysis

The demographic and clinical characteristics of the study and control groups were analyzed utilizing descriptive statistics and percentages. Differences in tinnitus severity between the groups were assessed using an independent *t*-test. The analysis of qEEG involved a mixed-design analysis of variance (ANOVA), evaluating the effects of eye condition (open vs. closed), the group (study vs. control), and their interaction (condition and group). Statistical significance was set at *p* ≤ 0.05. Analysis used IBM SPSS Statistics (v. 24).

## 3. Results

### 3.1. Hearing Thresholds

**Figure 1:**
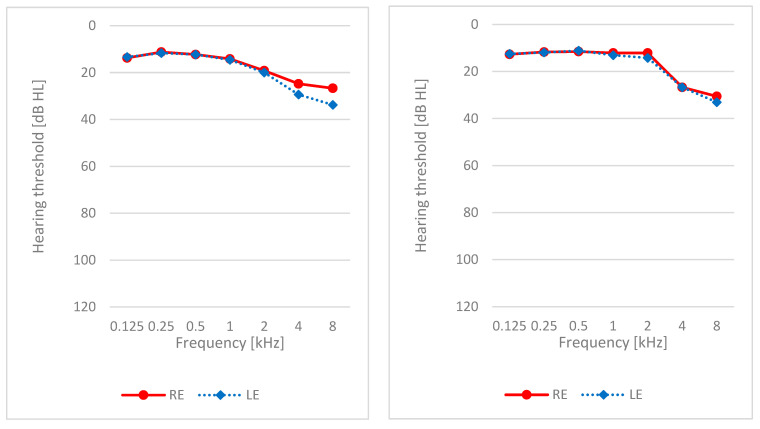
Hearing thresholds: study group (**left**), control group (**right**).

Average hearing thresholds across all frequencies (from 0.125 to 8 kHz) are shown in Figure 1. Average hearing thresholds were, for most frequencies, better than 20 dB HL, although they were slightly worse at higher frequencies, which is typical for age-related hearing loss. In the study group, there were 19 patients (79%) who had mean hearing thresholds better than 20 dB HL in the right ear and 16 (67%) who had mean hearing thresholds better than 20 dB HL in the left ear. In the control group, the figures were 18 (69%) and 17 (65%), respectively.

### 3.2. Tinnitus Characteristics

In both groups, the minimum duration of tinnitus was 0.5 years. Within the study group, 12 patients reported tinnitus in the left ear, 7 in the right, and 5 in both ears or in the head. On the other hand, in the control group, tinnitus was most frequently reported in both ears or in the head (16 patients), followed by 6 in the left ear and 4 in the right.

In the study group, patients experienced a dominant tinnitus pitch ranging from 1 to 12.5 kHz. The majority (n = 12) had a tinnitus pitch of 8 kHz or higher; 10 patients reported pitches between 4 and 6 kHz, and 2 below 3 kHz. Tinnitus loudness ranged from 5 to 20 dB SL (M = 10.9; SD = 3.9). There were 15 patients who characterized their tinnitus as tonal, while 9 described it as narrowband noise.

Within the control group, the tinnitus pitch varied between 0.125 and 16 kHz. The majority (n = 13) reported tinnitus pitch at 8 kHz or higher, while 4 patients reported pitches between 4 and 6 kHz, 4 between 3 and 1 kHz, and 5 below 1 kHz. Tinnitus loudness ranged from 5 to 50 dB SL (M = 12.7; SD = 9.0). There were 19 patients who described their tinnitus as tonal, 6 as narrowband noise, and 1 patient did not provide a characterization of their tinnitus.

### 3.3. Tinnitus Severity

Tinnitus severity, as assessed by the Tinnitus Handicap Inventory (THI), exhibited a comparable level between the study and control groups. Within the study group, THI scores ranged from 4 to 92 points, with a mean score of 45.4 (SD = 23.5). Likewise, in the control group, THI scores ranged from 14 to 94 points, with a mean score of 45.8 (SD = 23.2). There was no significant difference between the groups (t = 0.07; *p* = 0.947).

### 3.4. qEEG

Measures of bioelectrical activity at each electrode revealed a large and statistically significant increase in power when eyes were closed compared to eyes open, irrespective of group affiliation. This effect was particularly evident in the alpha and alpha 2 bands, although there were also observable impacts in the theta, low beta, middle beta, and beta 2 bands. There was no effect in the theta band. 

Looking at the effect of the group, there was no statistically significant difference between the groups for any electrode. 

However, we did find a statistically significant interaction effect between group and condition for one particular electrode, T3, over the auditory cortex. The effect was evident in several bands: alpha 2 (F = 4.57; *p* = 0.038; η^2^ = 0.09); theta (F = 5.66; *p* = 0.021; η^2^ = 0.11), low beta (F = 8.26; *p* = 0.006; η^2^ = 0.15), mid beta (F = 8.77; *p* = 0.005; η^2^ = 0.15), and beta 2 (F = 5.54; *p* = 0.023; η^2^ = 0.10). The differences are shown in Table 1.

Table 1 shows that, within the study group, bioelectrical activity on the T3 electrode was significantly lower in the eyes-closed condition than in the control group. This feature was evident in the alpha 2 (*p* = 0.048), low beta (*p* = 0.030), middle beta (*p* = 0.015), and beta 2 (*p* = 0.022) bands.

Furthermore, in the eyes-open condition, bioelectrical activity on the T3 electrode increased significantly compared to the eyes-closed condition, but only within the study group. This effect was prominent in the middle beta (*p* = 0.028) and beta 2 (*p* = 0.015) bands, with no discernible difference seen in the control group.

Conversely, within the control group, bioelectrical activity on the T3 electrode was significantly higher in the eyes-closed condition compared to the eyes-open condition, notably in the alpha 2 (*p* < 0.001), theta (*p* = 0.010), and low beta (*p* = 0.008) bands. Surprisingly, no such distinctions were observed in the study group, indicating a differential response between the two groups under these conditions.

## 4. Discussion

Building upon prior research demonstrating an association between tinnitus severity and symptoms of anxiety and depression [23], our study aimed to investigate the possible impact of the COVID-19 pandemic and the accompanying isolation on individuals suffering from tinnitus. We wanted to see whether the stressors during the pandemic period might have affected our subjects. Our approach was to compare two similar cohorts of tinnitus sufferers tested before and during the COVID-19 pandemic. We assumed that any difference might be attributed to the effects of stress induced by the pandemic and the associated isolation.

Contrary to expectations, the THI questionnaire showed no significant effects of the pandemic on self-reported tinnitus severity. At the same time, qEEG revealed notable reductions in electrical activity within the auditory cortex during the COVID-19 period, particularly in the alpha and beta bands. These changes reflect some sort of alteration in neurophysiology due to the pandemic and reinforce the intimate connection between external stressors and the neurobiology of tinnitus. The qEEG may have reflected early, pre-clinical changes that were not revealed by the THI questionnaire. Further research is needed to draw out the subtle dynamics at play and the implications for the tinnitus clinic.

A number of studies have seen an augmentation of the EEG alpha band in individuals with tinnitus compared to non-tinnitus controls [24,25,26,27]. At the same time, research on stress, albeit unrelated to COVID-19 specifically, has shown a general decrease in alpha activity as stress increases [28]. There are a few studies that have employed qEEG to investigate people’s response to COVID-19, although none have specifically looked at tinnitus. A comprehensive review by Kopańska et al. [29] found that the SARS-CoV-2 virus alters certain aspects of the nervous system detectable via an EEG. Another report also found a high prevalence of abnormal EEG background activity in COVID-19 patients [30]. One notable study reported a marked reduction in alpha and beta activity in the EEG of COVID-19 patients [31]. A related study saw a reduction in the peak frequency of alpha wave activity during isolation and a reversal of this effect when the isolation ended [17]. The current study follows on from these studies and contributes, in the context of the unique environment offered by the COVID-19 pandemic, to the literature on the interplay between tinnitus, stress, and the EEG alpha band. Our study also supports other research that recommends EEG as a means of evaluating tinnitus [32]. 

The reduction in alpha activity we observed in our tinnitus patients during the pandemic aligns broadly with findings from previous work. The decline in alpha appears to be associated with elevated stress levels and the effects of isolation induced by the pandemic. Such an observation is consistent with the study by Milner et al. [12], which compared EEGs recorded while the subject focused attention on their tinnitus with EEGs acquired during concentration on other internal sensations. In subjects with high distress due to tinnitus, Milner and colleagues saw a decrease in alpha band activity when tinnitus was the focus of attention. This suggests that during the COVID-19 isolation period, patients may have become more attuned to their tinnitus, which in turn led to a decrease in alpha. Although our study only included subjects who did not report COVID-19 infection, it is possible that some may have been infected unknowingly, and this may have had an impact on qEEG measures. 

It is important to emphasize that the picture of how EEGs relate to tinnitus is still far from clear. There are contradictory findings, particularly in terms of the alpha band. Whereas a majority of studies indicate increased alpha in tinnitus subjects [23,25,26], some have seen lower alpha levels in tinnitus subjects, levels which increased again after tinnitus remission [33,34].

In our study, we also saw a decrease in the beta EEG band. However, establishing a consistent picture within the existing literature is a challenge, as findings regarding beta are even less conclusive than those for alpha. Some studies have shown a parallel increase in beta and alpha, while others indicate a divergence, such as a reduction in alpha and an increase in beta as stress grows [35]. A systematic review of the effect of stress on the EEG concludes that the results for the beta band are inconclusive [27].

The COVID-19 pandemic and its requirements for isolation have introduced significant additional stress into the lives of individuals worldwide. Given the established link between tinnitus and stress [36], it is reasonable to expect that there would have been a negative impact on tinnitus sufferers at this time. The potential effect of COVID-19 infection on hearing loss adds another layer of complexity. There are reports of general hearing loss related to COVID-19 [37] as well as of certain other problems—for example, one preliminary report describes a higher incidence of Meniere’s disease during the COVID-19 pandemic [38]. At the same time, it is well known that hearing loss may lead to tinnitus. While systematic reviews conclude that there is a worsening of tinnitus due to the pandemic in general or from COVID infection in particular [6,7], all these authors highlight that the quality of studies is not always high, and some of them did not use control groups [7].

Nevertheless, the overall scale of tinnitus-related issues appears to have increased during the pandemic. Indirectly, we know that there was an increase in internet searches related to tinnitus during the COVID-19 period [39,40]. This suggests a significant increase in stress levels among tinnitus sufferers during COVID-19, even though some studies fail to support this. 

Taken together, the current body of evidence presents a mixed picture. Although some studies point to COVID-19 making things worse for tinnitus sufferers, there are also studies finding no such effect. For instance, a recent study conducted among the elderly found that 90% of participants experienced no change in tinnitus, and there were equal numbers who said their tinnitus worsened as well as improved [41].

As with our investigation, a range of studies have also used THI as a tool to explore the impact of the COVID-19 pandemic on tinnitus. One such study, by Beukes and colleagues, employed the screening version of THI to investigate 3103 tinnitus patients during the pandemic; it found that 32% experienced a worsening of tinnitus severity, whereas only 1% reported an improvement [42]. Additional studies conducted during the pandemic have also reported an increase in tinnitus [43,44]. 

However, more recent investigations present slightly more divergent findings. One study demonstrated a notable increase in tinnitus loudness, reduced ability to concentrate because of tinnitus, and higher THI scores, but only in patients who contracted COVID-19 [45]. Intriguingly, this study also observed a significant rise in hyperacusis among uninfected tinnitus patients. Another recent study found that lifestyle changes during the COVID-19 pandemic and subsequent lockdown did not significantly affect the severity of tinnitus [46]. Our present study aligns more closely with the latter, as we saw no significant increase in THI scores.

Finally, some limitations of the study should be mentioned. The study group was quite small, and a better approach would have been to test exactly the same group of subjects before and during the COVID-19 pandemic (like in [20]). Unfortunately, we did not have access to such data. The subject group sizes used here were similar to other EEG studies (e.g., [14,26]).

## 5. Conclusions

The current study gives a valuable perspective on the impact of the COVID-19 pandemic on self-reported measures of tinnitus severity. Notably, it stands as the first investigation to present resting-state EEG findings in tinnitus patients during the pandemic. The outcomes of the study align with some recent, albeit numerically small, studies that indicate no discernible effect of the pandemic on the subjective perception of tinnitus severity. Nevertheless, our work did observe alterations in electrical activity within the auditory cortex, manifested as a reduction in the amplitude of the prominent alpha rhythm. This suggests that the experience of isolation may have led to a degree of cortical deactivation, and this aspect deserves further investigation.

## Figures and Tables

**Table 1:** Bioelectrical activity measured at electrode T3 for the study and control groups. Results are listed for two experimental conditions (eyes open and closed). Only bands exhibiting significant differences are shown, in which there is an interaction between group and eye condition.

Band	Group	Eyes Open		Eyes Closed		
		M (SD)	Study vs. Control (*p*-Value)	M (SD)	Study vs. Control (*p*-Value)	Open vs. Closed (*p*-Value)
Alpha 2	Study group	0.70 (0.48)	n.s.	1.11 (1.07)	0.048	n.s.
	Control group	0.94 (1.55)		2.09 (2.13)		<0.001
Theta	Study group	0.94 (0.63)	n.s.	0.84 (0.65)	n.s.	n.s.
	Control group	0.79 (0.88)		1.16 (0.73)		0.010
Low beta	Study group	0.60 (0.41)	n.s.	0.48 (0.28)	0.030	n.s.
	Control group	0.49 (0.46)		0.73 (0.49)		0.008
Middle beta	Study group	0.48 (0.37)	n.s.	0.32 (0.19)	0.015	0.028
	Control group	0.39 (0.33)		0.52 (0.36)		n.s.
Beta 2	Study group	0.88 (0.92)	n.s.	0.51 (0.28)	0.022	0.015
	Control group	0.73 (0.62)		0.90 (0.75)		n.s.

M—mean, SD—standard deviation, n.s.—not significant.

## Data Availability

The data underlying this article are attached as a Supplementary File.

## Supplementary Materials

The following supporting information can be downloaded at: https://www.mdpi.com/article/10.3390/brainsci14020174/s1, data used for Figure 1, and Table 1.
